# Response to the letter to the editor: On the feasibility of dynamical analysis of network models of biochemical regulation

**DOI:** 10.1093/bioinformatics/btac318

**Published:** 2022-05-13

**Authors:** Felix M Weidner, Julian D Schwab, Silke D Werle, Nensi Ikonomi, Ludwig Lausser, Hans A Kestler

**Affiliations:** Institute of Medical Systems Biology, Ulm University, 89081 Ulm, Germany; International Graduate School of Molecular Medicine, Ulm University, 89081 Ulm, Germany; Institute of Medical Systems Biology, Ulm University, 89081 Ulm, Germany; Institute of Medical Systems Biology, Ulm University, 89081 Ulm, Germany; Institute of Medical Systems Biology, Ulm University, 89081 Ulm, Germany; International Graduate School of Molecular Medicine, Ulm University, 89081 Ulm, Germany; Institute of Medical Systems Biology, Ulm University, 89081 Ulm, Germany; Institute of Medical Systems Biology, Ulm University, 89081 Ulm, Germany

In his letter to the editor, Luis Rocha addresses the concern that other researchers might be discouraged from further investigation of dynamic analyses in Boolean networks based on a statement from our recently published manuscript (Weidner *et al.*, 2021). In particular, the author refers to a phrase in our abstract on the feasibility of dynamic investigations in large Boolean models. We value the discussion on this crucial topic in the field of Boolean networks. However, we kindly disagree on the interpretation of the respective parts of our manuscript.

First, we want to refer to the point addressed in our abstract. Here, we state: ‘However, since dynamic complexity of these models grows exponentially with their size, **exhaustive** analyses of the dynamics and consequently screening all possible interventions eventually becomes infeasible’. In addition, in our introduction, we report that: ‘Nevertheless, also for BNs, it holds that dynamic complexity scales exponentially with network size, again limiting the possibility of **complete** dynamic investigations’. This sentence comes with a reference explaining the feasibility of exhaustive attractor computation in Boolean network models. Here, we are entirely in line with the view given, and this is also what is elaborated throughout our manuscript.

Foremost, when it comes to screening potential interventions targets using Boolean networks, dynamic analyses in the sense of exhaustive screening become very complex with a growing number of compounds in the model and potential targets or even combinations of targets. Our method does not aim to be a replacement of dynamic analysis but a step in the screening for intervention targets, scaling down the number of interventions to screen. Subsequently, the identified targets can be evaluated by different perturbation analyses based on network dynamics, such as more detailed studies of the attractor landscape (Müssel *et al.*, 2010), or an automated screening (Schwab and Kestler, 2018). Especially when adding another layer of complexity, such as with large reconstructed networks or even populations of those (Schwab *et al.*, 2021), detailed intervention screening on top of attractor evaluation becomes complex, and interaction graph-based pre-screening methods become helpful.

*Financial Support*: none declared.

*Conflict of Interest*: none declared.
